# Time for the lens to focus on diagnostic algorithms in asthma

**DOI:** 10.1016/j.eclinm.2024.102812

**Published:** 2024-09-04

**Authors:** Richard Beasley, Jonathan Noble

**Affiliations:** a Medical Research Institute of New Zealand, Wellington, New Zealand; b Victoria University Wellington, Wellington, New Zealand

The lack of a gold standard diagnostic test for asthma contributes to the fundamental problem of both underdiagnosis and overdiagnosis. It has been estimated from population-based studies that between 20 and 70% of people with asthma in the community remain undiagnosed and hence untreated.^1^ On the opposite side of the spectrum, studies of patients with physician-diagnosed asthma suggest that 30–35% of adults and children diagnosed with asthma do not have current asthma, suggesting that asthma is also over diagnosed in the community. The consequences of this scenario do not just relate to the lack of treatment in those with asthma in whom the diagnosis has not been made. Those misdiagnosed with asthma will not only suffer from the consequences of receiving incorrect treatment for their actual disorder, but asthma treatment may also result in systemic adverse effects from inhaled and/or oral corticosteroid treatment, progressively escalated due to the lack of a therapeutic response. Correcting a misdiagnosis is complex, in part because treatment potentially confounds future diagnostic tests. This can lead to a challenging clinical conundrum of identifying an incorrect diagnosis, in whom treatment should be withdrawn, versus a poor responder, in whom treatment should be escalated. The importance of this dilemma has led to consensus guidelines groups developing diagnostic algorithms for use in clinical practice.

The validity of diagnostic algorithms and their potential utility have been brought into sharp focus by Andrew Simpson and colleagues with their recent prospective observational study of adults referred with clinician-suspected asthma who had undergone multiple investigations and a trial of inhaled corticosteroids.^2^ Published in this edition of the journal, their analyses show that the current Global Initiative for Asthma (GINA)^3^ and United Kingdom's National Institute for Health and Care Excellence (NICE)^4^ asthma diagnostic algorithms provide excellent specificity but low sensitivity, whereas the European Respiratory Society (ERS)^5^ diagnostic algorithms have only reasonable sensitivity and specificity, when compared with the reference diagnosis determined by the panel of asthma experts. In determining whether an individual had asthma, the panel reviewed all the clinical information, including results of the extensive investigations such as spirometry, fractional exhaled nitric oxide (FeNO), peak flow variability, bronchodilator challenge testing with methacholine and mannitol, and response to both bronchodilator and inhaled corticosteroid (ICS) treatment. The provision of such extensive information provides confidence in their ‘gold-standard’ diagnosis and interpretation that the GINA and NICE guidelines afford excellent utility for ruling in asthma, but limited ability to rule it out, and the ERS guidelines would lead to a problematic degree of uncertainty. Furthermore, more than one third of adults could have been given a different diagnosis depending on which guideline algorithm was followed. The predictive properties of the individual investigations were provided, informing their potential inclusion in future algorithms.

The logical diagnostic process starts with a clinical history and examination. The characteristic signs and symptoms of asthma have modest sensitivity and specificity for asthma, increasing with combinations of symptoms, especially in children.^6^ This supports the concept that the presence of progressively more characteristic features of asthma, the greater the probability of asthma, as proposed by the British Thoracic Society.^6^ However, there is the need to enhance the diagnostic accuracy, and there are three general approaches that can be employed.

One approach is to undertake one or more specific investigations such as measurement of spirometry, bronchodilator reversibility, peak flow variability and FeNO, all of which have high specificity but low/modest sensitivity. As most cases of suspected asthma present to primary care, potential algorithms need to be flexible and pragmatic, to enable them to be used in such settings in which not all diagnostic tests are available.

Secondly, an essential part of any diagnostic algorithm is to systematically consider the presence of overlapping disorders such as chronic obstructive pulmonary disease, bronchiectasis and inducible laryngeal obstruction in adults, that may present with asthma-like symptoms in an individual without asthma, or in combination with asthma.^7^^,^^8^ Identifying such ‘treatable traits’ reduces the risk of an asthma misdiagnosis and inappropriate treatment, while also enabling individualised treatment.

The third approach is a trial of inhaled corticosteroids for an individual with suspected asthma, which has high specificity for asthma, as reported in this study.^2^

The first step to resolving a clinical conundrum, is to identify that there is a problem, so that targeted research can be undertaken. The authors have highlighted the limitation of the current diagnostic algorithm approach to asthma, appropriately propose that new algorithms are needed, provide comment as to how these might be developed, and that they should be tested prospectively before implementation. The time has come for the lens to focus on diagnostic algorithms in asthma.

## Contributors

RB and JN made equal contributions to the writing of this editorial.

## Declaration of interests

No financial support was received for this manuscript. RB has received institutional research funding from AstraZeneca, Teva, HRC (NZ), Cure Kidz (NZ) and Perpetual Guardian; consulting fees from AstraZeneca, Avillion and Teva; honoraria for presentations from AstraZeneca; is Chair of the Asthma Foundation of New Zealand adolescent and adult asthma guidelines, reviewer for GINA and ex-member of the GOLD Board; and has received medication and monitors for clinical trials from AstraZeneca. JN has no conflicts of interest to declare.
